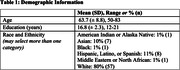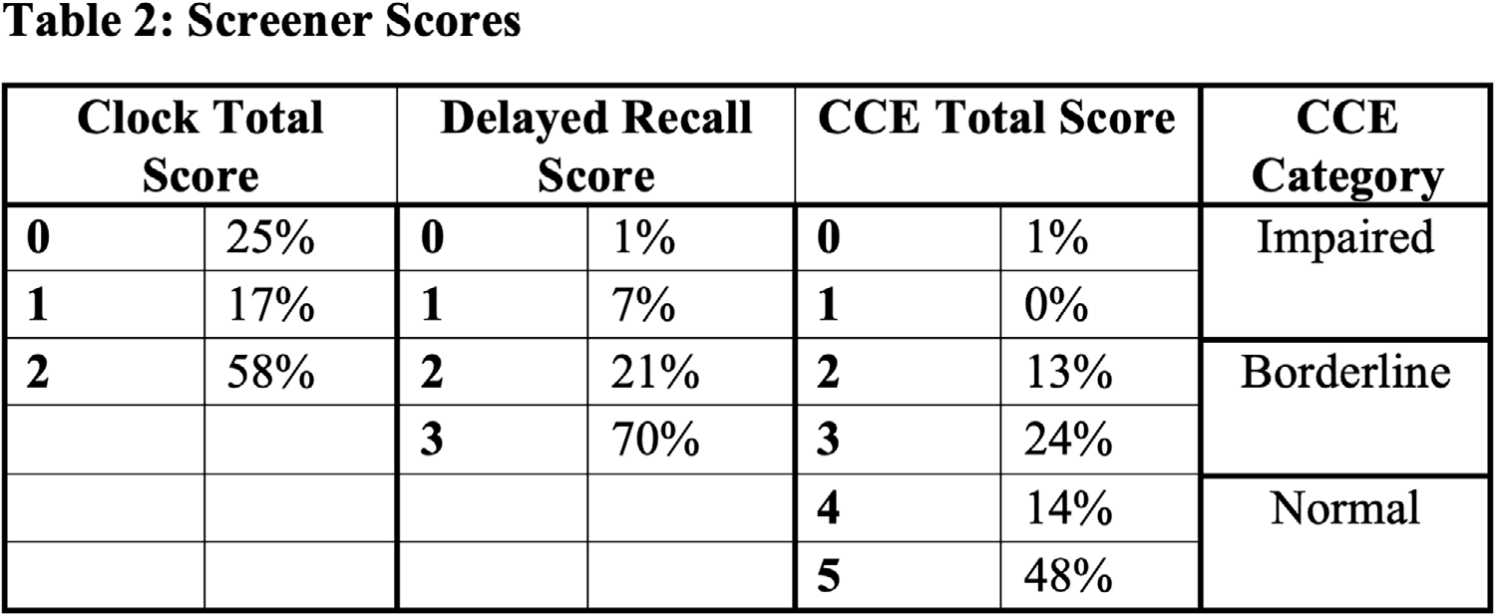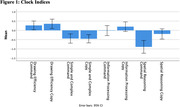# Digital Cognitive Screening Outcomes in Older Women at the Annual Well Woman Visit

**DOI:** 10.1002/alz.092636

**Published:** 2025-01-03

**Authors:** Sandra Rizer, Jillian L. Joyce, Samantha A. Dargie, Silvia Chapman, David J. Libon, Laura Mora, Sarah E. Tom, Karen Marder, Mary Rosser, Stephanie Cosentino

**Affiliations:** ^1^ Columbia University Irving Medical Center, New York, NY USA; ^2^ Rowan University, Stratford, NJ USA

## Abstract

**Background:**

Mild cognitive impairment, a precursor to Alzheimer’s disease and related disorders (ADRD), is widely underdiagnosed. Routine screenings are key for identifying older adults with emerging neurodegenerative disease. As women have increased risk of ADRD and often use their gynecologist as their primary care physician, the annual well woman visit offers a critical opportunity to screen older women for ADRD. This study aimed to examine objective cognitive outcomes using a novel digital screening tool easily deployed in clinical settings.

**Method:**

Women 50 and older were invited to participate in the current study at the time of their annual visit to the Columbia University Integrated Women’s Health Center. Participants completed a brief tablet‐based cognitive screener, the Core Cognitive Evaluation (CCE), developed by Linus Health. The CCE includes an immediate and delayed three‐word recall (3 points) and clock drawing (2 points) for a maximum of 5 points (Normal>3, Borderline 2‐3, Impaired<2), based on both core and error/process variables. A continuous 100‐point clock score is also produced with eight indices.

**Result:**

Of 130 eligible participants, 71(55%) agreed to participate in the study (see Table 1 for demographics). Continuous clock scores ranged from 16 to 100 (Mean(SD) = 73.15(21.2)). As described in Table 2, 62% participants scored in the Normal range, 37% in the Borderline range and 1% in the Impaired range. After a delay, 76% of participants recalled all or more of the words initially recalled. Lower scores were observed in clock drawing than delayed word recall, with spatial reasoning being the lowest the clock index score on average. See Figure 1 for clock indices.

**Conclusion:**

The majority of women obtained Normal CCE scores while 38% performed in the Borderline or Impaired range. By capturing subtle information about the process used to complete cognitive tasks, tablet‐based testing offers a sensitive means of detecting cognitive changes. Future work is needed to evaluate screener outcomes in relation to comprehensive neuropsychological testing and cognitive change over time. A limitation of this study is the sociodemographic homogeneity of the participants. Future work aims to expand into gynecology settings with more diverse patient populations.